# Using machine learning to combine genetic and environmental data for maize grain yield predictions across multi-environment trials

**DOI:** 10.1007/s00122-024-04687-w

**Published:** 2024-07-23

**Authors:** Igor K. Fernandes, Caio C. Vieira, Kaio O. G. Dias, Samuel B. Fernandes

**Affiliations:** 1 Department of Crop, Soil, and Environmental Sciences, Center for Agricultural Data Analytics, University of Arkansas, Fayetteville, AR USA; 2 Department of Crop, Soil, and Environmental Sciences, University of Arkansas, Fayetteville, AR USA; 3 Department of General Biology, Federal University of Viçosa, Viçosa, Brazil

## Abstract

**Key message:**

**Incorporating feature-engineered environmental data into machine learning-based genomic prediction models is an efficient approach to indirectly model genotype-by-environment interactions.**

**Abstract:**

Complementing phenotypic traits and molecular markers with high-dimensional data such as climate and soil information is becoming a common practice in breeding programs. This study explored new ways to combine non-genetic information in genomic prediction models using machine learning. Using the multi-environment trial data from the Genomes To Fields initiative, different models to predict maize grain yield were adjusted using various inputs: genetic, environmental, or a combination of both, either in an additive (genetic-and-environmental; G+E) or a multiplicative (genotype-by-environment interaction; GEI) manner. When including environmental data, the mean prediction accuracy of machine learning genomic prediction models increased up to 7% over the well-established Factor Analytic Multiplicative Mixed Model among the three cross-validation scenarios evaluated. Moreover, using the G+E model was more advantageous than the GEI model given the superior, or at least comparable, prediction accuracy, the lower usage of computational memory and time, and the flexibility of accounting for interactions by construction. Our results illustrate the flexibility provided by the ML framework, particularly with feature engineering. We show that the feature engineering stage offers a viable option for envirotyping and generates valuable information for machine learning-based genomic prediction models. Furthermore, we verified that the genotype-by-environment interactions may be considered using tree-based approaches without explicitly including interactions in the model. These findings support the growing interest in merging high-dimensional genotypic and environmental data into predictive modeling.

**Supplementary Information:**

The online version contains supplementary material available at 10.1007/s00122-024-04687-w.

## Introduction

Genotype-by-environment interaction (GEI) plays an essential role in plant breeding, resulting in differential changes in individual performance or rank-changing across environments (Falconer 1996; Tabery 2008; Bernardo 2014). Consequently, prediction frameworks that do not consider GEI have been shown to underperform in multi-environment trials (MET) (Burgueño et al. 2012; Jarquín et al. 2017; Gillberg et al. 2019). One approach often used in MET is the linear mixed model with a factor analytic structure modeling the variance-covariance between environments (Smith et al. 2001; de los Campos and Gianola 2007; Dias et al. 2018). Another alternative proposed based on the mixed model framework is to incorporate environmental data with a reaction norm model utilizing covariance structures that account for the genetic similarity between genotypes and the similarity among environmental conditions (Jarquín et al. 2014). These models were necessary steps toward developing a MET prediction framework. However, they are still limited in their utilization of environmental data due to the constraint of incorporating GEI only through covariance structures, which limits, for instance, the inclusion of high-order interactions in the model.

The high availability of environmental data in testing locations has catalyzed a thorough characterization of environmental effects over the observed phenotype. In addition to the traits commonly measured by plant breeders, data from weather stations, soil surveys, and public repositories have been recently integrated into genomic prediction (GP) models (Malosetti et al. 2016; Monteverde et al. 2019; Canella Vieira et al. 2022). These environmental data can be applied in enviromics studies by envirotyping the testing locations (Costa-Neto et al. 2021, 2022) or in a combined way with biological knowledge through crop growth models to increase prediction accuracy in GP (Heslot et al. 2014; Technow et al. 2015). In this case, increases of up to 11% in prediction accuracy have been observed (Heslot et al. 2014).

One approach that has been gaining momentum when modeling GEI is machine learning (ML) (Montesinos-López et al. 2019; Jubair et al. 2023). The flexibility of integrating high-dimensional and multilayered data makes ML a good alternative for plant breeding, especially as weather, soil, and other environmental information become more commonly used in GP models. The ML model can leverage this diversity of data to improve the learning process, resulting in higher prediction accuracy (Gong et al. 2019). How this information is processed to derive new features (i.e., the feature engineering stage) may also play an essential role in the model’s performance. However, applications of ML in GP are recent, and currently, there is no consensus on the best ML approach to combine environmental and genetic data when accounting for GEI.

Some of the recent ML methods utilized in the GEI context include neural networks for predicting yield, protein content, and oil content (Ray et al. 2023), convolutional neural networks (CNN) to predict grain yield using genetic, environmental, management, and historical (e.g. yield and weather) data (Washburn et al. 2021) and dense neural networks (DNN) using intermediate layers to allow interactions between the different data sets (Kick et al. 2023). Tree-based ML models such as Random Forest (Breiman 2001), XGBoost (Chen and Guestrin 2016), and LightGBM (Ke et al. 2017) are other examples of successful applications of ML in modeling GEI. However, none of these studies extensively explored feature engineering, which could be an effective approach to including environmental data in GP models. We hypothesized that by using feature engineering, we would be able to efficiently characterize the environment (i.e., envirotyping), which, combined with additive and non-additive genetic data, would result in high prediction accuracy. Therefore, we propose GP models that use feature-engineered environmental data, additive and non-additive genotypic data, or a combination of both. Our findings indicate that combining genotypic and environmental data in ML using our approach is an efficient strategy for predicting maize grain yield in multi-environment trials.

## Materials and methods

### Phenotypic information

This study used the MET data set from the Genomes to Fields (G2F) 2022 Maize Genotype by Environment Prediction Competition (Genomes to Fields 2023; Lima et al. 2023). Specifically, we used the trial information from 2019, 2020, and 2021, which consisted of 1,179 maize (*Zea mays L.*) hybrids evaluated in 72 environments (a combination of year and location), comprising 14 states in the USA (CO, DE, GA, IA, IL, IN, MI, MN, NC, NE, NY, SC, TX, and WI) and one city in Germany (Göttingen).

Trials from 2019 included two testers (LH195, PHT69), and trials from 2020 and 2021 included four testers (LH195, PHZ51, PHK76, and PHP02). The experimental design used in the trials was a modified Randomized Complete Block Design (RCBD), mainly with two replications per environment. The response variable used in this study consisted of grain yield in ${\rm Mg}~{\rm ha}^{-1}$ at 15.5% grain moisture. More details on 2019, 2020, and 2021 genetic material are available at Lopez-Cruz et al. (2023).

**Fig. 1:**
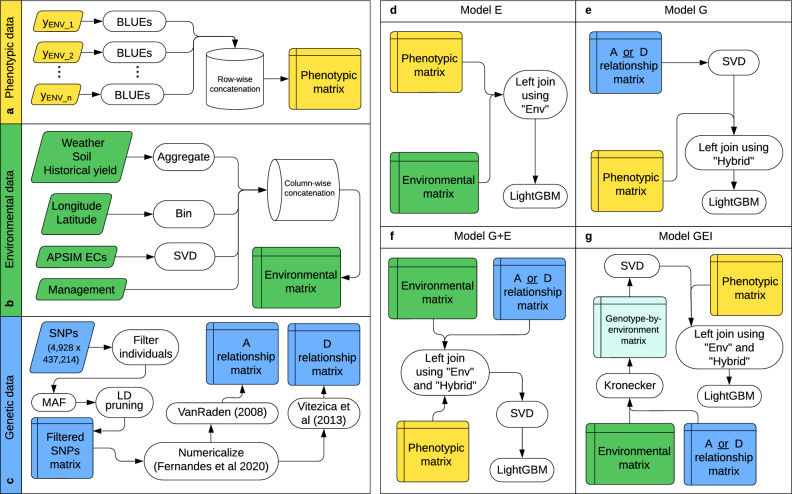
Workflow for machine learning genomic prediction. Data preprocessing steps (panels **a**–**c**) and model fitting steps (panels **d**–**g**). **a**, $\text {y}_{\rm ENV_1}$, $\text {y}_{\rm ENV_2}$, $\dots$, $\text {y}_{\rm ENV_n}$ represent the response variable vector for the $1 {\rm st}$, $2 {\rm nd}$, $\dots$, $n {\rm th}$ environments. Single-environment trial models are adjusted for each environment using equation (1) to compute the Best Linear Unbiased Estimates (BLUEs). Then, the BLUEs are concatenated row-wise to form the phenotypic matrix, which contains the columns “Env” (environment), “Hybrid” (hybrid), and “y” (BLUE). The columns “Env” and “Hybrid” are used as primary keys in further left join operations. **b**, Each type of environmental data proceeds to a different preprocessing step: weather, soil, and historical yield data are aggregated based on various summary statistics to produce new features, longitude and latitude are binned to reduce the noise around the varying locations, Agricultural Production Systems sIMulator (APSIM) environmental covariates (ECs) are moved to a Singular Value Decomposition (SVD) step to reduce their dimensionality from 765 to 15 columns, yet explaining 99.9% of the variance, and management is used as is. All the resulting features are then concatenated column-wise to create the environmental matrix, which includes the column “Env” (environment), used as a primary key in further left join operations. **c**, The matrix of single nucleotide polymorphisms (SNPs), consisting of 4928 individuals and 437,214 markers, proceeds to several steps for filtering and quality control: filtering to keep only individuals phenotyped in 2019, 2020, and 2021, excluding SNPs with minor allele frequency (MAF) less than 0.01, and pruning SNPs based on a Linkage Disequilibrium (LD) threshold of $r^2 =$ 0.9, a window size of 100, and a step size of 20. The resulting matrix retains 1179 individuals and 67,083 markers. Then, this matrix is passed to a numericalization step to convert genotypes to numbers (Fernandes and Lipka 2020) and moves to the calculation of genomic relationship matrices. Using VanRaden (2008) yields the additive (A) genomic relationship matrix, and Vitezica et al. (2013) produces the dominance (D) genomic relationship matrix, in which both matrices have dimensions $1179 \times 1179$. **d**, E: environmental. The phenotypic and environmental matrices are combined through a left join using the “Env” column as the primary key and then fitted to the LightGBM framework. **e**, G: genetic. The genomic relationship matrix (A or D) is passed to an SVD step to keep only 100 components, merged to the phenotypic matrix via left join using the “Hybrid” column as the primary key, and then fitted to the LightGBM framework. **f**, G+E: genetic-and-environmental. The environmental matrix, the genomic relationship matrix (A or D), and the phenotypic matrix are connected through a left join using the “Env” and “Hybrid” columns as primary keys. The environmental and genetic features are passed to an SVD step to keep only 100 components, and the resultant data is fitted to the LightGBM framework. **g**, GEI: genotype-by-environment interaction. A Kronecker product between the environmental matrix and the genomic relationship matrix (A or D) is taken to form the genotype-by-environment matrix, which proceeds to an SVD step to keep only 100 components, linked to the phenotypic matrix via a left join using the “Env” and “Hybrid” columns as primary keys, and then fitted to the LightGBM framework

Single-environment trial models, adjusted using the package ASReml-R 4 (Butler et al. 2017), were fitted to generate the best linear unbiased estimates (BLUEs) for each hybrid in each environment as follows:

1 $$\begin{aligned} Y_{ijklm} = \mu + H_i + R_j + b(R)_{jk} + r_l + c_m + e_{ijklm} \end{aligned}$$

where $Y_{ijklm}$ is the observed grain yield of the $i^{th}$ hybrid of the $j^{th}$ replicate, $k^{th}$ block, $l^{th}$ row, and $m^{th}$ column; $\mu$ is the intercept; $H_i$ is the fixed effect of the $i^{th}$ hybrid; $R_j$ is the fixed effect of the $j^{th}$ replicate; $b(R)_{jk}$ is the random effect of the $k^{th}$ block nested within the $j^{th}$ replicate with $b(R)_{jk} \sim N(0, \sigma ^2_b)$; $r_l$ is the random effect of the $l^{th}$ row, with $r_l \sim N(0, \sigma ^2_r)$; $c_m$ is the random effect of the $m^{th}$ column, with $c_m \sim N(0, \sigma ^2_c)$; and $e_{ijklm}$ is the residual term associated with the $ijklm^{th}$ experimental unit, with $e_{ijklm} \sim N(0, \sigma ^2_e)$. BLUEs from model 1 were used as the response variable for further analysis (Fig. 1a).

For each location in 2019, 2020, and 2021, the coefficient of variation was calculated as follows:

2 $$\begin{aligned} \text {CV} = \frac{\sqrt{\sigma ^2_r}}{\mu _{\hat{y}}} \times 100 \end{aligned}$$

where $\sqrt{\sigma ^2_r}$ and ${\mu _{\hat{y}}}$ are the square root of the residual variance component and the mean of predicted values, respectively, from the equation (1).

The generalized heritability (Cullis et al. 2006) for each location in 2019, 2020, and 2021 was calculated as follows:

3 $$\begin{aligned} H^2_{\ \text {Cullis}} = 1 - \frac{\bar{V} (\Delta )}{2\sigma ^2_g} \end{aligned}$$

where ${\sigma ^2_g}$ is the genetic variance component, and $\bar{V} (\Delta )$ is the mean pairwise prediction error variance. To estimate the heritability, we used a similar model as (1), where $H_i$ was treated as a random effect.

### Environmental information

Each location was equipped with a Spectrum WatchDog 2700 weather station, which collected information on variables such as rainfall, solar radiation, humidity, and air temperature every 30 min daily. Aggregations were employed to derive new environmental features. Within each environment, weather data was aggregated based on the season and various summary statistics were calculated, including the mean, maximum, minimum, and standard deviation of each weather variable (Supplementary Table S1).

Similarly, lagged yield features were created based on the historical grain yield. For each field location, we calculated summary statistics such as the mean, minimum, and percentiles of the grain yield in the previous year (Supplementary Table S1), i.e., when the observed yield was from 2021, these features were calculated based on the grain yield of 2020 for a given field location. When the observed yield was from 2020, grain yield data from 2019 was used instead.

In some environments, the field trials were close to each other. We used latitude and longitude to obtain bins representing nearby regions to reduce the noise around the varying locations. Bins were obtained as follows:

4 $$\begin{aligned} x' = \Bigl \lfloor \frac{x}{s} \Bigr \rfloor \times s \end{aligned}$$

where $x'$ is the new binned latitude or longitude, $\lfloor . \rfloor$ is the floor operator, *x* is the latitude or longitude to be binned, and *s* is a step parameter to control the binning range. The greater the *s*, the lower the number of unique latitude and longitude values created. For example, latitudes equal to 39.785, 39.824, and 39.927 would all become 39.6 when using $s=1.2$. We used $s=1.2$ and $s=3.6$ as step parameters for latitude and longitude, respectively.

The data set also included 765 environmental covariates (ECs) derived using an Agricultural Production Systems sIMulator (APSIM) crop model from an unpublished work available in (Lima et al. 2023) (see file “COMPETITION_DATA_README.docx” at https://doi.org/10.25739/tq5e-ak26). Briefly, this model used $200 {\rm kg}~{\rm ha}^{-1}$ of NO_3_ fertilization at all locations, and phenological periods were estimated based on averages from the training set and are not specific to any hybrid. The ECs are formed by a combination of a variable, a phenological period, and a soil layer. Soil layers are specified as 1 through 10 (1 is the top layer), with each layer consisting of 20 cm in the soil column for a total of two meters deep. For example, the EC denoted as “SDR_pGerEme_1” is based on the variable Water Supply–Demand Ratio (“SDR”) during the phenological period from germination to emergence (“pGerEme”) within the soil layer from 0 to 20 cm below ground (soil layer “1”). All the variables and phenological periods utilized are available in the aforementioned file.

As there was a large number of ECs, we performed dimensionality reduction using a Singular Value Decomposition (SVD), utilizing the package scikit-learn 1.2.1 (Pedregosa et al. 2011) from Python 3.8 (Van Rossum and Drake 2009). After performing SVD, we kept the first 15 components, which explained 99.9% of the variance.

Finally, we also used soil variables such as available Nitrates in parts per million, amount of Nitrogen in pounds per acre, and percentage of calcium. In total, we ended up with 90 environmental features stored in a matrix called the environmental matrix, where each row represents a unique environment, and each column represents an environmental feature. The 90 environmental features comprised 6 categories: 63 weather-related, 15 derived from the ECs after dimensionality reduction, 6 derived from the lagged historical grain yield, three soil-based, two based on geographical coordinates, and one related to management (Supplementary Table S1). Details of the data preprocessing steps are illustrated in Fig. 1b.

### Genetic information

The genotypic data was described in Lima et al. (2023). Briefly, variant calls for the 2014–2023 G2F materials were obtained using the Practical Haplotype Graph (PHG) (Bradbury et al. 2022). Hybrid genotypes were generated by combining information about their parent lines using the CreateHybridGenotypes plugin available in TASSEL 5 (Bradbury et al. 2007), which yielded a file with 4,928 individuals and 437,214 markers. We used VCFtools 0.1.15 (Danecek et al. 2011) to keep only the individuals evaluated in 2019, 2020, and 2021, which resulted in a data set with 1,179 individuals. Next, we excluded SNPs with minor allele frequency (MAF) less than 0.01 using VCFtools. Finally, we conducted a step of Linkage Disequilibrium (LD) pruning using PLINK 1.9 (Purcell et al. 2007) with the option “–indep-pairwise”, which is based on the squared Pearson’s correlation coefficient ($r^2$), along with a window size of 100, step size of 20, and $r^2$ threshold of 0.9. The final file consisted of 1,179 hybrids and 67,083 markers, which was converted to a numeric format using the package simplePHENOTYPES 1.3.0 (Fernandes and Lipka 2020) to enable us to calculate genomic relationship matrices.

We used the package AGHmatrix (Amadeu et al. 2023) from R 4.2.2 (R Core Team 2023) to create additive (A) (VanRaden 2008) and dominance (D) (Vitezica et al. 2013) relationship matrices. Thus, the genetic information utilized for downstream analysis consisted of the A matrix ($1179 \times 1179$) and the D matrix ($1179 \times 1179$) (see Fig. 1c for an illustration of the genetic data preprocessing steps).

### Genomic prediction models

All genomic prediction models were fitted with a Gradient Boosting Machine (GBM) model implemented in the LightGBM (Ke et al. 2017) framework from Python 3.8. The models utilized different inputs for training, and they were grouped into four categories, namely, environmental (E), genetic (G), genetic-and-environmental (G+E), and genotype-by-environment interaction (GEI). All models using genetic information were independently fitted with A or D genomic relationship matrices. All models included the field location (i.e., the environment name without the year) as a categorical variable.

#### Environmental (E)

The E model was fitted using the data set of 90 environmental features (Supplementary Table S1). This data set was connected to the phenotypic data through a left join using the “Env” (environment) column as the primary key and fitted to the LightGBM framework. As these 90 environmental features were calculated using only the environmental data, all their values are the same within each environment regardless of the hybrid. Therefore, all predictions are the same within a given environment. Figure 1d outlines the E model fitting steps.

#### Genetic (G)

The G model was adjusted using the genomic relationship matrices previously obtained. The G models are denoted as G(A) when using the A matrix and G(D) when using the D matrix. The first step before using the genomic relationship matrix is to obtain the first 100 components from the SVD (Supplementary Table S2). Next, these components are merged to the phenotypic data via a left join using the “Hybrid” (hybrid) column as the primary key to be fitted to the LightGBM framework (Fig. 1e).

#### Genetic-and-environmental (G+E)

The G+E model integrates genotypic and environmental information but does not include an explicit genotype-by-environment interaction effect. The possible G+E models are denoted as G(A)+E when using the A matrix and G(D)+E when using the D matrix. The environmental and genetic data were connected to the phenotypic data through a left join using the “Env” and “Hybrid” columns as primary keys, where the environmental and genetic features together are decomposed with the SVD to obtain the first 100 components (Supplementary Table S2). The resultant data was fitted to the LightGBM framework (Fig. 1f).

#### Genotype-by-environment interaction (GEI)

For fitting GEI models, we calculated Kronecker products between the environmental matrix and a given genomic relationship matrix as follows:

5 $$\begin{aligned} {\textbf{X}}_{nq \times mq} = {\textbf{E}}_{n \times m} \otimes {\textbf{G}}_{q \times q} \end{aligned}$$

where ${\textbf{X}}$ is the new genotype-by-environment matrix,^1^${\textbf{E}}$ is the environmental matrix with *n* unique environments and *m* environmental features, and ${\textbf{G}}$ is the genomic relationship matrix (either A or D) with *q* unique hybrids. Thus, the possible GEI models are denoted as G(A)EI when using the A matrix and G(D)EI when using the D matrix.

The ${\textbf{X}}$ matrix proceeded to an SVD step using the first 100 components (Supplementary Table S2). Next, these components are combined with the phenotypic data through a left join using the “Env” and “Hybrid” columns as primary keys and fitted to the LightGBM framework (Fig. 1g). As the genotype-by-environment matrix (${\textbf{X}}$) contains both environment and hybrid information, a unique prediction is obtained for each hybrid $\times$ environment combination.

#### Factor analytic multiplicative mixed model (FA)

To compare our results with a commonly used genomic prediction model, we fitted a Factor Analytic Multiplicative Mixed Model (FA) using the package ASReml-R 4 (Butler et al. 2017). The BLUEs of each hybrid $\times$ environment combination were used as the response variable when fitting the following statistical model:

6 $$\begin{aligned} Y_{ij} = \mu + E_i + h(E)_{ij} + e_{ij} \end{aligned}$$

where $Y_{ij}$ is the BLUE of the $j^{th}$ hybrid on the $i^{th}$ environment; $\mu$ is the intercept; $E_i$ is the fixed effect of the $i^{th}$ environment; $h(E)_{ij}$ is the random effect of the $j^{th}$ hybrid within the $i^{th}$ environment, with $h(E)_{ij} \sim N(0, \varvec{\Sigma }_g)$, $\varvec{\Sigma }_g = \textbf{FA}_1 \otimes {\bf{G}}$, where $\textbf{FA}_1$ is the factor analytic matrix of order 1 of environments, ${\bf{G}}$ is the additive genomic relationship matrix of hybrids, $\otimes$ is the Kronecker product, and $e_{ij}$ is the residual term associated with the $ij^{th}$ experimental unit, with $e_{ij} \sim N(0, \sigma ^2_e)$.

### Cross-validation schemes

We performed three cross-validation (CV) schemes as done in Sukumaran et al. (2018). All the schemes used ten repetitions of k-fold cross-validation with $k=5$. Thus, in all cases, the phenotypic information was divided into five subsets, and each subset was used once as the validation set, while the remaining four were used as the training set.

#### CV2

The CV2 scheme was concerned with predicting the performance of known hybrids in known environments but with unknown combinations of hybrid and environments (sparse testing).

The following steps were taken for each of the five folds: (1) sample 20% of all the environment-hybrid combinations to build the validation set, and (2) remove this 20% randomly chosen combinations from the training set (trials from 2020 and 2021).

#### CV1

The CV1 scheme involved predicting the performance of unknown hybrids in known environments. The models were trained using trials from 2020 and 2021, and the prediction was made using trials from 2021.

The steps taken for each of the five folds were: (1) sample 20% of all hybrids from 2021 trials to build the validation set, and (2) remove this 20% randomly chosen hybrids from the training set (trials from 2020 and 2021).

#### CV0

The CV0 scheme predicted the performance of known hybrids in a new year. The models were trained using data from 2019 and 2020, and the predictions were made on 2021 trials.

The procedure applied for each of the five folds was as follows: (1) sample 20% of the hybrids evaluated in 2021 to build the validation set; (2) take the phenotypic data from 2019 and 2020 for these 20% randomly chosen hybrids to include in the training set, and (3) sample an additional 60% hybrids evaluated either in 2019 or 2020 trials and add to the training set. This last step was done to obtain an 80 : 20 proportion for the training and validation sets as in the other cross-validation schemes utilized in this study.

### Metrics

As usually done in GP studies, we used Pearson’s correlation coefficient between the observed and the predicted yield to assess the prediction accuracy of the models. Another metric utilized was the Coincidence Index (CI) (Hamblin and Zimmermann 1986), which calculates how well the top predicted hybrids overlap with the top observed hybrids. The Coincidence Index (CI) was calculated considering the top 20% hybrids as follows:

7 $$\begin{aligned} \text {CI} = \frac{B - R}{T - R} \end{aligned}$$

where *T* is the total number of hybrids to evaluate, *B* is the number of overlapping hybrids (i.e., hybrids common to both observed and prediction sets), and *R* is the expected number of hybrids selected by chance. The CI was another metric employed in this study because it provides an alternative to compare how different models perform in terms of a predefined percentage of selected individuals.

Prediction accuracy pairwise comparisons were conducted following Meng et al. (1992) to assess model performance differences. For example, the pairwise comparison “FA - G(A)+E” denotes the difference between Pearson’s correlation of BLUEs and predictions of the FA model and Pearson’s correlation of BLUEs and predictions of the G(A)+E model. This type of statistical test compares two overlapping correlations based on dependent groups because both correlations share one variable in common (i.e., the BLUEs) and the same set of samples. A significance level of 0.05 was adopted, and the Bonferroni correction was applied to reduce the type I error.

The mean proportion of overlapping testers between training and validation populations was calculated as follows:

8 $$\begin{aligned} \bar{p} = \frac{1}{kr} \sum _{i=1}^k \sum _{j=1}^{r} \frac{|W_{ij} \cap Z_{ij}|}{|W_{ij} \cup Z_{ij}|} \end{aligned}$$

where $\bar{p}$ is the mean proportion of overlapping testers between training and validation populations, *i* is the $i^{th}$ fold, $i = \{1, \dots , 5\}$, *j* is the $j^{th}$ repetition, $j = \{1, \dots , 10\}$, ${|W_{ij} \cap Z_{ij}|}$ is the number of overlapping testers between training (*W*) and validation (*Z*) sets, and ${|W_{ij} \cup Z_{ij}|}$ is the number of testers in training and validation sets.

### Code and data availability

A repository containing all the scripts and documentation on reproducing the results is available at https://github.com/igorkf/Maize_GxE_Prediction. All the initial data used in this study are available at https://doi.org/10.25739/tq5e-ak26 (Genomes to Fields 2023; Lima et al. 2023). All intermediate files and scripts as well as final predictions obtained from our models are deposited at https://zenodo.org/records/12702650. All the plots were created using the packages matplotlib 3.2.2 (Hunter 2007) and seaborn 0.12.2 (Waskom 2021).

## Results

The average coefficient of variation across years was 10.8%, 17.1%, and 15.1%, with values ranging from 0.1% to 22.3%, 11.5% to 27.9%, and 9.0% to 26.7% across environments, in 2019, 2020, and 2021, respectively (Supplementary Figure S1).

The average heritability across years was 0.38, 0.39, and 0.47, with values ranging from 0 to 0.84, 0.01 to 0.63, and 0 to 0.82 across environments, in 2019, 2020, and 2021, respectively (Supplementary Figure S1).

**Fig. 2:**
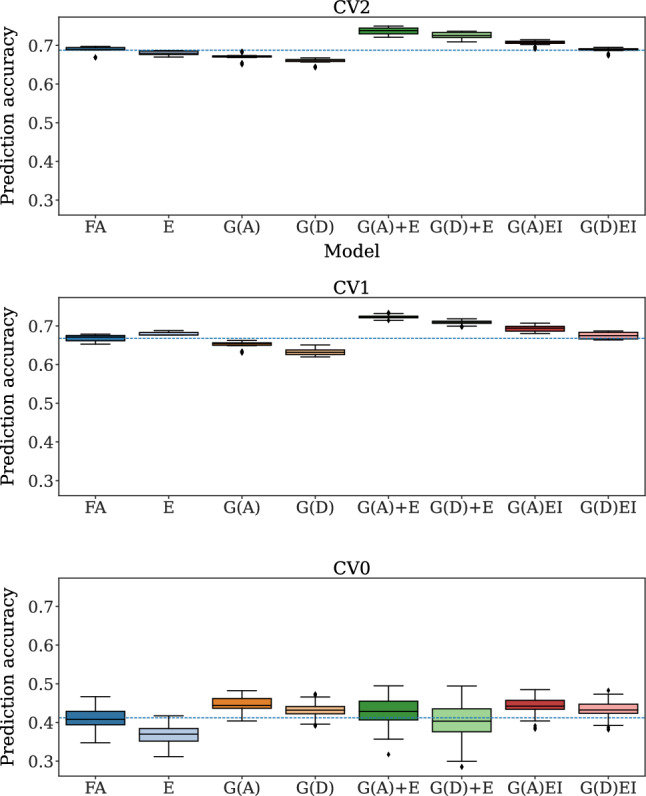
Prediction accuracy (the Pearson’s correlation between the observed and the predicted yield) of 5-fold cross-validation (CV) over ten repetitions for each model and CV scheme. The dashed line represents the mean prediction accuracy of the Factor Analytic Multiplicative Mixed Model (FA). G(.), G(.)+E, and G(.)EI are the genetic, genetic-and-environmental, and genotype-by-environment interaction models, respectively, with (.) being a genomic relationship matrix (A: additive or D: dominance)

A great variation was also observed in prediction accuracy across models, especially when comparing different CV schemes. The mean prediction accuracy across CV schemes was 0.69, 0.68, and 0.42, with values ranging from 0.64 to 0.75, 0.62 to 0.73, and 0.28 to 0.49 across models in CV2, CV1, and CV0, respectively (Fig. 2).

**Fig. 3:**
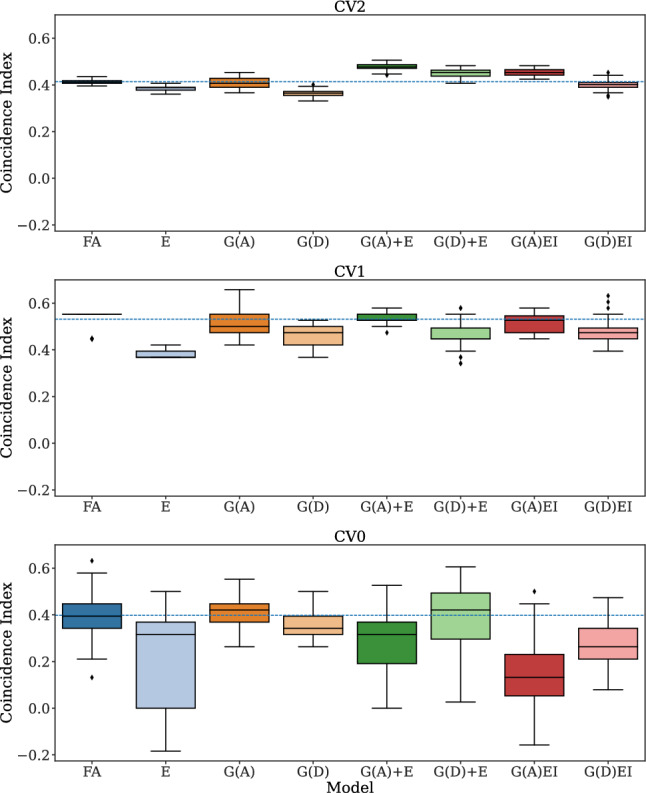
Coincidence Index (CI) of 5-fold cross-validation (CV) over ten repetitions for each model and CV scheme. The dashed line represents the mean CI of the Factor Analytic Multiplicative Mixed Model (FA). G(.), G(.)+E, and G(.)EI are the genetic, genetic-and-environmental, and genotype-by-environment interaction models, respectively, with (.) being a genomic relationship matrix (A: additive or D: dominance). The CI was calculated considering the top 20% hybrids. This metric is used to compare how different models perform in terms of a predefined percentage of selected individuals

Likewise, the mean CI showed variation across CV schemes (0.42, 0.48, and 0.31), with values ranging from 0.33 to 0.51, 0.34 and 0.66, and  − 0.18 to 0.63 across models in CV2, CV1, and CV0, respectively (Fig. 3).

### Population structure

**Fig. 4:**
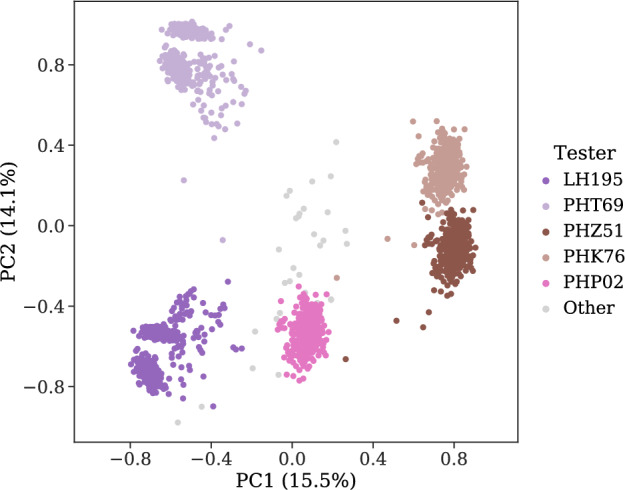
Principal component analysis (PCA) of the population structure. The first two principal components (PC1 and PC2) explain 29.6% of the variance. The relative frequency of hybrids per tester is 23.7% (LH195), 23.9% (PHT69), 17.2% (PHZ51), 16.9% (PHK76), 16.7% (PHP02), and 1.6% (Other)

Six groups were identified from a Principal Component Analysis (PCA), with the first two principal components explaining 29.6% of the variance. Using eq. (8), we noticed that, on average, a proportion of 100%, 90%, and 72% of overlapping testers was used between training and validation populations for the CV2, CV1, and CV0 schemes, respectively (Fig. 4).

### Depending on the cross-validation scheme, the genetic data alone can result in the worst prediction

For CV2 and CV1 schemes, the models that only used genetic information resulted in the worst performance in terms of prediction accuracy (Fig. 2).

For CV2, the prediction accuracy was statistically different among FA and G(D) (means 0.69 and 0.66) and among FA and G(A) (means 0.69 and 0.67). In both pairwise comparisons, FA exhibited better performance (Supplementary Table S3).

Likewise, for CV1, the prediction accuracy statistically differed between FA and G(D) (means 0.67 and 0.63) and between FA and G(A) (means 0.67 and 0.65), where, in both cases, FA showed better performance (Supplementary Table S3).

For CV0, the prediction accuracy ranged from 0.40 to 0.48 for model G(A) and from 0.39 to 0.47 for model G(D) with means 0.45 and 0.43, respectively. In contrast, FA resulted in a lower mean prediction accuracy of 0.41, with values ranging from 0.35 to 0.47. The pairwise comparisons FA and G(A) and FA and G(D) were statistically significant (Supplementary Table S3).

The models with only genetic information, particularly the dominance one, also showed a low coincidence index (CI) performance under CV2 and CV1 schemes (Fig. 3).

While the FA model had a mean CI of 0.41, the mean CI values for models G(D) and G(A) resulted in a prediction accuracy of 0.36 and 0.41, respectively, under the CV2 scheme.

Similarly, for CV1, the mean CI values were 0.46 for G(D), 0.52 for G(A), and 0.53 for FA.

For the CV0 scheme, G(A) showed the best mean CI (0.41), with values ranging from 0.26 to 0.55, while the model G(A)EI performed the worst with a mean CI of 0.14 and values ranging from − 0.16 to 0.50 (Fig. 3).

With the inclusion of environmental information in the G models, the mean prediction accuracy was at least equal to the FA model for all the CV schemes. For example, all G+E models yielded higher mean prediction accuracy than FA in CV1 and CV2 (Fig. 2). Furthermore,

G(A)+E had a significant boost in mean prediction accuracy over G(A) of $10.0\%$ for CV2 and $11.1\%$ for CV1 (Supplementary Table S3).

In the CV0 scenario, the models G(A)+E and G(A)EI both resulted in a mean prediction accuracy of 0.45, which is $7.1\%$ greater than the mean prediction accuracy achieved by the FA, with both differences being statistically significant. Overall, regarding prediction accuracy, the G(A)+E model was superior or at least equal to all other models across all the CV schemes evaluated in this study (Supplementary Table S3).

## Discussion

The success of modern breeding programs lies not only in the scale of data acquisition but also in the different types of information used in the decision-making process. In this study, we investigated the inclusion of several non-genetic types of data in genomic prediction models. We incorporated all of these data by taking advantage of the flexibility of machine learning models such as GBM. We compared them with a standard approach utilized for MET analysis.

Many trials were performed in the G2F initiative from 2019 to 2021. Each environment employed different sets of hybrids to compose the yield trials. In total, 1,179 unique hybrids were evaluated in this study, although many of the hybrids were not used in all the environments (Supplementary Figure S2). The number of common hybrids among environments shows significant variability, and few hybrids from the 2019 trials were tested in the 2020 and 2021 trials.

### The influence of the genetic and environmental diversity in the prediction models

The genetic diversity of the population and the relatedness between the training and validation sets are critical factors in genomic prediction accuracy (Crossa et al. 2017; Fraslin et al. 2022). The number of testers used in the G2F initiative population changed over the years. For the subset of the data utilized in our research, two testers were used in 2019 and four testers in 2020 and 2021 (one of the testers from 2019 and three new testers). The genetic diversity was mostly driven by these five testers utilized (Fig. 4). Conversely, the magnitude of the environmental variability was considerably higher than what is typically observed in breeding programs, given that the environments include very distinct locations. The larger diversity of the environment compared with the genetic diversity could be the reason for the environmental model, which predicts the average of a given environment for all hybrids, to perform similarly to genomic models.

### The importance of environmental information should not be underestimated

Several studies have indicated that environmental information helps to enhance the prediction of phenotypic performance (Heslot et al. 2014; Technow et al. 2015; Costa-Neto et al. 2021, 2022). When modeling GEI in maize with machine learning, Westhues et al. (2021) did not observe much gain when predicting plant height, but the grain yield prediction improved after including environmental information. The prediction accuracies from CV1 and CV2 indicate that, on average, the environmental model was just as good as the genetic models. However, accurately selecting the top individuals is more relevant than average prediction for breeding programs. As expected, using environmental variables alone was not as useful in selecting the top 20% individuals. However, in the G+E combination, the selection of the top 20% individuals was still equal or superior to all models evaluated. Our results are further evidence that environmental data should not be neglected in prediction models, especially when considering the G+E scenario.

### The usefulness of feature engineering will depend on the approaches utilized

Several options exist for doing the feature engineering step in time series data, such as weather. We summarised weather conditions using the season as the grouping factor, which differed from previous studies in maize (Westhues et al. 2021; Kick et al. 2023). Although Westhues et al. (2021) utilized feature engineering without much success they derived features based on developmental stages. The authors argued that, due to phenotyping costs, the crop developmental stages through time were incorporated in their models using only three main developmental stages (vegetative, flowering, and grain filling), which could have negatively affected the efficiency of their features. We did not attempt to use the growing stages as a grouping factor. Kick et al. (2023) utilized k-means with dynamic time warping to cluster the weather and management time-series data, which enhanced the performance of models that only used genomic data. Also, we included the field location as a categorical factor in all the ML models to account for constant environmental effects (e.g., soil texture, management practices, etc.).

### The usefulness of diverse data for accurate prediction

Including several data types in ML is often more straightforward than in linear models for GP. Because of this flexibility, our models benefited from utilizing a diverse set of information, including historical yield, ECs, soil properties, and climatic and geographical information. Although no attempt was made to fit individual models with each of the different types of data available, the initial results observed in the model fitting process (data not shown) indicated an advantage of keeping all the environmental information utilized in our final models. The advantages of utilizing historical information, for instance, have also been observed in linear models for GP (Rutkoski et al. 2015; Dias et al. 2020). However, we use it in a fundamentally different way as we utilize the historical yield from hybrids that are not necessarily related to the target hybrids to characterize each environment. ECs are another type of data utilized in our model that is becoming more common in GP models (Bustos-Korts et al. 2019; Onogi 2022; Jighly et al. 2022). Our results confirmed that ECs such as the ones derived from the APSIM crop model are useful features in improving prediction abilities. Thus, developing approaches that directly integrate crop growth models with GP could be the best alternative for improving prediction accuracy. In agreement with what has been observed in the literature (Westhues et al. 2021; Washburn et al. 2021; Kick et al. 2023), our results indicate that researchers should consider broadening GP to phenotypic prediction based on multiple types of data.

### GEI for machine learning

Our GEI models used Kronecker products to explicitly create interactions between each hybrid in the genomic relationship matrix and each environment in the derived environmental matrix. However, the Kronecker product abruptly increases the dimensionality of the data set, and fitting models using the resulting data set is not feasible. When using the GEI models, the number of columns in the resulting data set is massive ($>100,000$). Moreover, the memory consumption for GEI models surpassed 200GB of RAM, which is usually available only at high-performance computing (HPC) clusters. Conversely, the G+E models were much more parsimonious and computationally efficient, given that the number of columns in the data set is around 1250–1300. Despite having much smaller dimensionality than GEI models, the G+E models still had many features, which increases the time for model fitting and the chance of overfitting, given that not all features are individually meaningful. Thus, to overcome the problem of high dimensionality with possible redundancy of information and large model-fitting time, we employed the SVD method to reduce the data set dimensionality before fitting the models (Fig. 1e, f, g; Supplementary Table S2).

As noted by Westhues et al. (2021), tree-based machine learning models do not require an explicit inclusion of genotype-by-environment interaction as input to the model, given that a high-order interaction between features is captured by construction (Friedman 2001). This fact was corroborated in this study, where the G+E model, which only integrates genetic and environment data through the concatenation of data sets, was better or at least similar to the performance of GEI models in all the CV schemes. The G+E model was much more parsimonious than the GEI model and was computationally efficient, requiring approximately 30 s to fit the model for one fold and one repetition (data not shown) on a general-purpose computational node with two Intel(R) Xeon(R) Gold 6130 CPU @ 2.10GHz processors, equipped with 32 cores and 192GB of RAM. This contributes to the need for more efficient computational strategies for integrating genomic and environmental data to expand GP models to new environments and germplasm, enhancing our comprehension of genotype-by-environment interactions (Rogers and Holland 2021).

## Conclusion

This study demonstrates the massive importance of the environment in the outcome of a prediction model. As a means to incorporate such information into genomic prediction models, the ML framework offers great flexibility, especially when utilizing feature engineering. Our results illustrate that the feature engineering step presents a valuable envirotyping option, creating useful variables for ML-based genomic prediction models. As the amounts and diversity of data available in breeding programs increase, there will be more opportunities for utilizing feature engineering in breeding programs. We demonstrated that environmental features, in combination with genetic data, can significantly improve prediction accuracy over models using only genetic information. Furthermore, we confirmed that with tree-based methods, the genotype-by-environment interactions can be accounted for without explicitly including interactions in the model. Collectively, these results are promising, especially with the increasing interest in combining envirotyping and genotyping approaches for prediction purposes.

### Supplementary Information

Below is the link to the electronic supplementary material.

Supplementary file 1 (pdf 196 KB)

## Abbreviations

ML: Machine learning
G+E: Genetic-and-environmental
GEI: Genotype-by-environment interaction
FA: Factor analytic multiplicative mixed model
MET: Multi-environment trials
GP: Genomic prediction
CNN: Convolutional neural networks
DNN: Dense neural networks
G2F: Genomes to fields
RCBD: Randomized complete block design
BLUEs: Best linear unbiased estimates
ECs: Environmental covariates
APSIM: Agricultural production systems sIMulator
SVD: Singular value decomposition
PHG: Practical haplotype graph
SNP: Single-nucleotide polymorphism
MAF: Minor allele frequency
LD: Linkage disequilibrium
A: Additive
D: Dominance
GBM: Gradient boosting machine
E: Environmental
G: Genetic
CV: Cross-validation
CI: Coincidence index
PCA: Principal component analysis
HPC: High-performance computing
PAR: Photosynthetically active radiation

## Footnotes

1 Because of its large size, the ${\textbf{X}}$ matrix was saved in the “arrow” format using the package arrow 12.0.0 (Richardson et al. 2023). This format was adopted for fast reading the ${\textbf{X}}$ matrix before fitting the models, given that the model needed to be fitted many times (i.e., across different cross-validation (CV) schemes, and repetitions).
