# Supplementary material for: Using machine learning to combine genetic and environmental data for maize grain yield predictions across multi-environment trials
Source: Theor Appl Genet. 2024 Jul 23;137(8):189. doi: 10.1007/s00122-024-04687-w (PMC11266441; doi:10.1007/s00122-024-04687-w)
Supplement: Supplementary file 1 — (pdf 196 KB) [file 122_2024_4687_MOESM1_ESM.pdf]

Appendix

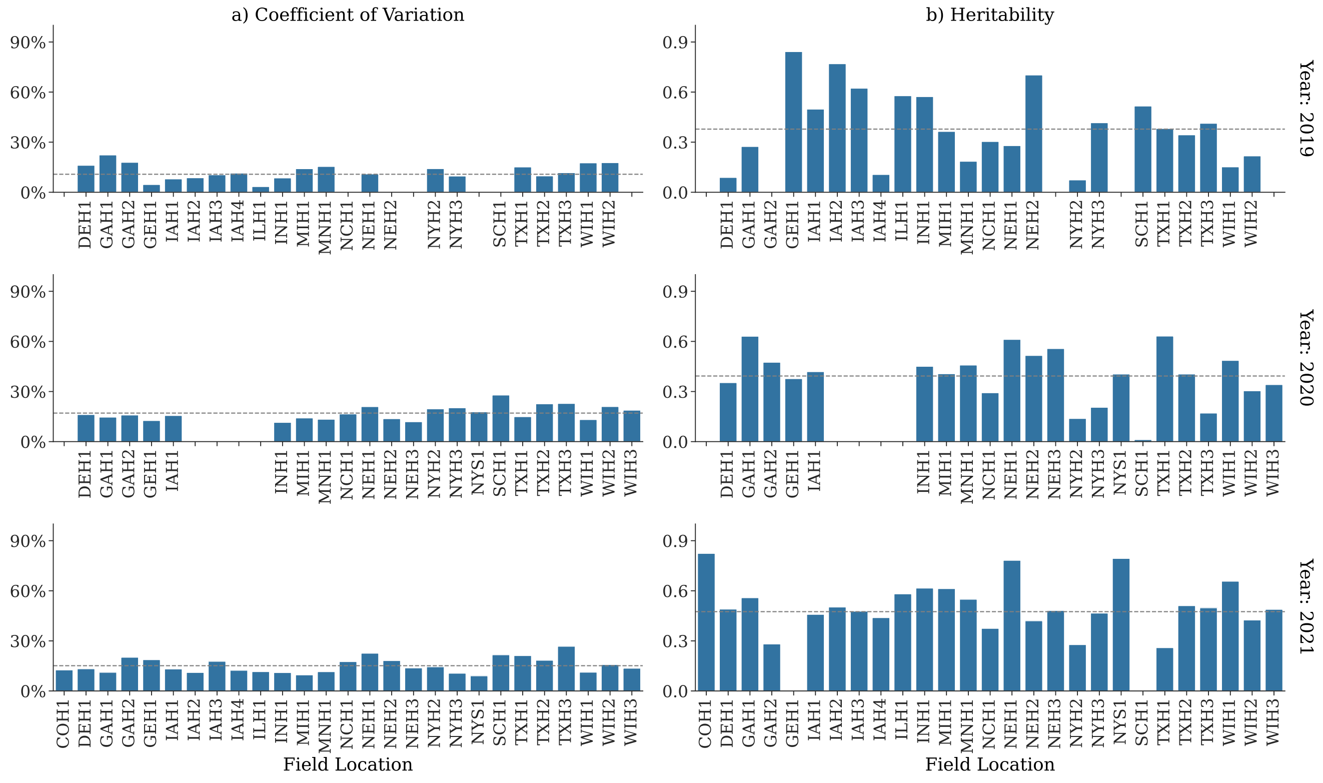

Fig. S1: Coefficient of Variation and Heritability for each location for 2019, 2020, and 2021 trials. The grey dashed lines represent the mean value across locations within each year. Some locations were absent or removed from the study for some years.

Table S1: Description of all the environmental features. Std. dev. is the standard deviation.

Feature	Description
weather_station_lat	The vertical global position of the weather station
weather_station_lon	The horizontal global position of the weather station
treatment_not_standard	Whether the treatment was not standard (e.g., drought, irrigated, etc.)
T2M_max_fall	Maximum temperature (in °C) at 2 meters in fall
T2M_max_spring	Maximum temperature (in °C) at 2 meters in spring
T2M_max_summer	Maximum temperature (in °C) at 2 meters in summer
T2M_max_winter	Maximum temperature (in °C) at 2 meters in winter
T2M_min_fall	Minimum temperature (in °C) at 2 meters in fall
T2M_min_spring	Minimum temperature (in °C) at 2 meters in spring
T2M_min_summer	Minimum temperature (in °C) at 2 meters in summer
T2M_min_winter	Minimum temperature (in °C) at 2 meters in winter
T2M_std_fall	Standard deviation of temperature (in °C) at 2 meters in fall
T2M_std_spring	Standard deviation of temperature (in °C) at 2 meters in spring
T2M_std_summer	Standard deviation of temperature (in °C) at 2 meters in summer
T2M_std_winter	Standard deviation of temperature (in °C) at 2 meters in winter
T2M_mean_fall	Mean temperature (in °C) at 2 meters in fall
T2M_mean_spring	Mean temperature (in °C) at 2 meters in spring
T2M_mean_summer	Mean temperature (in °C) at 2 meters in summer
T2M_mean_winter	Mean temperature (in °C) at 2 meters in winter
T2M_MIN_max_fall	Maximum temperature (in °C) at 2 meters minimum in fall
T2M_MIN_max_spring	Maximum temperature (in °C) at 2 meters minimum in spring
T2M_MIN_max_summer	Maximum temperature (in °C) at 2 meters minimum in summer

Table S1 continued from previous page

Feature	Description
T2M_MIN_max_winter	Maximum temperature (in °C) at 2 meters minimum in winter
T2M_MIN_std_fall	Standard deviation of temperature (in °C) at 2 meters minimum in fall
T2M_MIN_std_spring	Standard deviation of temperature (in °C) at 2 meters minimum in spring
T2M_MIN_std_summer	Standard deviation of temperature (in °C) at 2 meters minimum in summer
T2M_MIN_std_winter	Standard deviation of temperature (in °C) at 2 meters minimum in winter
T2M_MIN_cv_fall	Coefficient of variation of temperature (in °C) at 2 meters minimum in fall
T2M_MIN_cv_spring	Coefficient of variation of temperature (in °C) at 2 meters minimum in spring
T2M_MIN_cv_summer	Coefficient of variation of temperature (in °C) at 2 meters minimum in summer
T2M_MIN_cv_winter	Coefficient of variation of temperature (in °C) at 2 meters minimum in winter
WS2M_max_fall	Maximum wind speed (in m/s) at 2 meters in fall
WS2M_max_spring	Maximum wind speed (in m/s) at 2 meters in spring
WS2M_max_summer	Maximum wind speed (in m/s) at 2 meters in summer
WS2M_max_winter	Maximum wind speed (in m/s) at 2 meters in winter
RH2M_max_fall	Maximum relative humidity (in %) at 2 meters in fall
RH2M_max_spring	Maximum relative humidity (in %) at 2 meters in spring
RH2M_max_summer	Maximum relative humidity (in %) at 2 meters in summer
RH2M_max_winter	Maximum relative humidity (in %) at 2 meters in winter
RH2M_p90_fall	90th percentile of relative humidity (in %) at 2 meters in fall
RH2M_p90_spring	90th percentile of relative humidity (in %) at 2 meters in spring
RH2M_p90_summer	90th percentile of relative humidity (in %) at 2 meters in summer
RH2M_p90_winter	90th percentile of relative humidity (in %) at 2 meters in winter
QV2M_mean_fall	Mean specific humidity (in g/kg) at 2 meters in fall
QV2M_mean_spring	Mean specific humidity (in g/kg) at 2 meters in spring
QV2M_mean_summer	Mean specific humidity (in g/kg) at 2 meters in summer
QV2M_mean_winter	Mean specific humidity (in g/kg) at 2 meters in winter
PRECTOTCORR_max_fall	Maximum precipitation corrected (in mm/day) in fall
PRECTOTCORR_max_spring	Maximum precipitation corrected (in mm/day) in spring
PRECTOTCORR_max_summer	Maximum precipitation corrected (in mm/day) in summer
PRECTOTCORR_max_winter	Maximum precipitation corrected (in mm/day) in winter
PRECTOTCORR_median_fall	Median precipitation corrected (in mm/day) in fall
PRECTOTCORR_median_spring	Median precipitation corrected (in mm/day) in spring
PRECTOTCORR_median_summer	Median precipitation corrected (in mm/day) in summer
PRECTOTCORR_median_winter	Median precipitation corrected (in mm/day) in winter
PRECTOTCORR_n_days_less_10_mm_fall	Number of days with precipitation corrected (in mm/day) less than 10mm in fall
PRECTOTCORR_n_days_less_10_mm_spring	Number of days with precipitation corrected (in mm/day) less than 10mm in spring
PRECTOTCORR_n_days_less_10_mm_summer	Number of days with precipitation corrected (in mm/day) less than 10mm in summer
PRECTOTCORR_n_days_less_10_mm_winter	Number of days with precipitation corrected (in mm/day) less than 10mm in winter
ALLSKY_SFC_PAR_TOT_std_fall	Standard deviation of all-sky surface total PAR (in W/m^2) in fall
ALLSKY_SFC_PAR_TOT_std_spring	Standard deviation of all-sky surface total PAR (in W/m^2) in spring
ALLSKY_SFC_PAR_TOT_std_summer	Standard deviation of all-sky surface total PAR (in W/m^2) in summer
ALLSKY_SFC_PAR_TOT_std_winter	Standard deviation of all-sky surface total PAR (in W/m^2) in winter
Nitrate_N_ppm_N	Available Nitrates in parts per million
lbs_N_A	Amount of Nitrogen in pounds per acre
percentage_Ca_Sat	Percentage of Calcium
EC_svd_comp0	0th component of environmental covariates after Singular Value Decomposition
EC_svd_comp1	1st component of environmental covariates after Singular Value Decomposition
EC_svd_comp2	2nd component of environmental covariates after Singular Value Decomposition
EC_svd_comp3	3rd component of environmental covariates after Singular Value Decomposition
EC_svd_comp4	4th component of environmental covariates after Singular Value Decomposition
EC_svd_comp5	5th component of environmental covariates after Singular Value Decomposition
EC_svd_comp6	6th component of environmental covariates after Singular Value Decomposition
EC_svd_comp7	7th component of environmental covariates after Singular Value Decomposition
EC_svd_comp8	8th component of environmental covariates after Singular Value Decomposition
EC_svd_comp9	9th component of environmental covariates after Singular Value Decomposition
EC_svd_comp10	10th component of environmental covariates after Singular Value Decomposition
EC_svd_comp11	11th component of environmental covariates after Singular Value Decomposition
EC_svd_comp12	12th component of environmental covariates after Singular Value Decomposition
EC_svd_comp13	13th component of environmental covariates after Singular Value Decomposition
EC_svd_comp14	14th component of environmental covariates after Singular Value Decomposition
mean_yield_lag_2	Mean yield of previous year within a field location
min_yield_lag_2	Minimum yield of previous year within a field location
p1_yield_lag_2	1st percentile of yield on the previous year within a field location

Table S1 continued from previous page

Feature	Description
q1_yield_lag_2	1st quartile of yield on the previous year within a field location
q3_yield_lag_2	3rd quartile of yield on the previous year within a field location
p90_yield_lag_2	90th percentile of yield on the previous year within a field location
T2M_std_spring_X_weather_station_lat	Std. dev. of temperature (in °C) at 2 meters in spring × vertical global position of weather station
T2M_std_fall_X_weather_station_lat	Std. dev. of temperature (in °C) at 2 meters in fall × vertical global position of weather station
T2M_min_fall_X_weather_station_lat	Minimum temperature (in °C) at 2 meters in fall × vertical global position of weather station

Table S2: Variance explained by the first 100 components of a Singular Value Decomposition (SVD) for each cross-validation (CV) scheme and model.

CV	Model	Variance explained
2	G(A)	99.9%
	G(D)	95.6%
	G(A)+E	99.9%
	G(D)+E	99.9%
	G(A)EI	99.6%
	G(D)EI	94.0%
1	G(A)	99.9%
	G(D)	95.7%
	G(A)+E	99.9%
	G(D)+E	99.9%
	G(A)EI	99.6%
	G(D)EI	94.1%
0	G(A)	99.8%
	G(D)	97.1%
	G(A)+E	99.9%
	G(D)+E	99.9%
	G(A)EI	99.6%
	G(D)EI	97.4%

Table S3: Prediction accuracy pairwise comparisons. Each contrast compares the performance of two different models on the same set of validation samples. A significance level of 0.05 was adopted, and the Bonferroni correction was applied. Estimate values are shown rounded to two decimals.

	CV2		CV1		CV0	
Contrast	Estimate	p-value	Estimate	p-value	Estimate	p-value
FA - E	0.01	n.s.	-0.01	n.s.	0.04	*
FA - G(A)	0.02	*	0.02	*	-0.03	*
FA - G(D)	0.03	*	0.03	*	-0.02	*
FA - G(A)+E	-0.05	*	-0.06	*	-0.03	*
FA - G(D)+E	-0.04	*	-0.04	*	-0.01	n.s.
FA - G(A)EI	-0.02	*	-0.03	*	-0.03	*
FA - G(D)EI	0.00	n.s.	-0.01	n.s.	-0.02	*
E - G(A)	0.01	n.s.	0.03	*	-0.08	*
E - G(D)	0.02	*	0.04	*	-0.06	*
E - G(A)+E	-0.06	*	-0.04	*	-0.08	*
E - G(D)+E	-0.05	*	-0.03	*	-0.06	*
E - G(A)EI	-0.03	*	-0.02	*	-0.08	*
E - G(D)EI	-0.01	*	0.00	n.s.	-0.06	*
G(A) - G(D)	0.01	*	0.02	*	0.02	*
G(A) - G(A)+E	-0.07	*	-0.07	*	0.00	n.s.
G(A) - G(D)+E	-0.06	*	-0.06	*	0.02	*
G(A) - G(A)EI	-0.04	*	-0.04	*	0.00	n.s.
G(A) - G(D)EI	-0.02	*	-0.03	*	0.01	*
G(D) - G(A)+E	-0.08	*	-0.09	*	-0.01	*
G(D) - G(D)+E	-0.07	*	-0.07	*	0.00	n.s.
G(D) - G(A)EI	-0.05	*	-0.06	*	-0.01	*
G(D) - G(D)EI	-0.03	*	-0.04	*	0.00	n.s.
G(A)+E - G(D)+E	0.01	*	0.01	*	0.02	*
G(A)+E - G(A)EI	0.03	*	0.03	*	0.00	n.s.
G(A)+E - G(D)EI	0.05	*	0.05	*	0.01	*
G(D)+E - G(A)EI	0.02	*	0.01	*	-0.02	*
G(D)+E - G(D)EI	0.04	*	0.03	*	-0.01	n.s.
G(A)EI - G(D)EI	0.02	*	0.02	*	0.01	*

p-value <0.05 is denoted as *; n.s. indicates a non-significant difference.

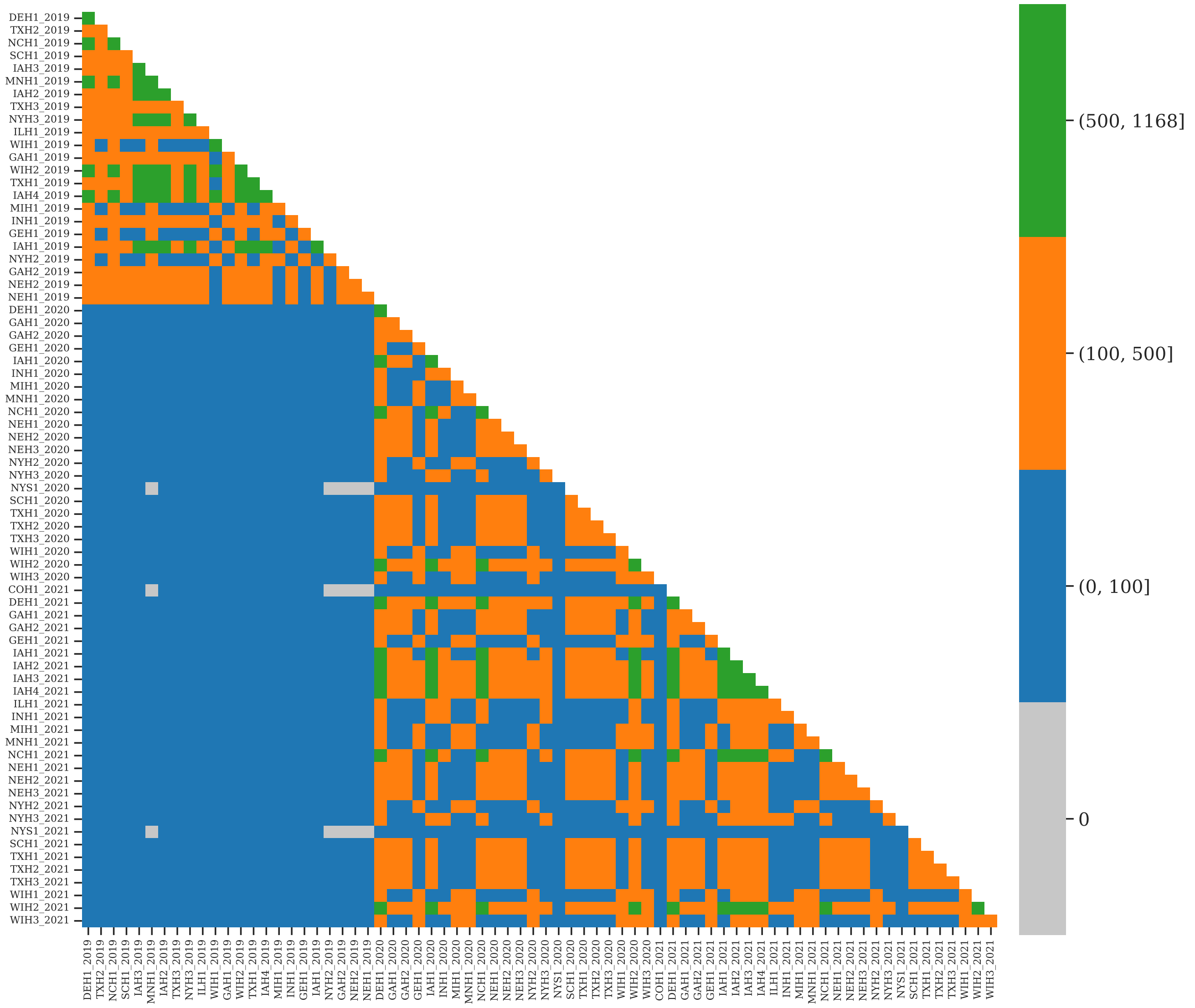

Fig. S2: Co-occurrence matrix of hybrids among environments. The diagonal values represent the number of unique hybrids in a given environment, whereas off-diagonal values represent the number of unique common hybrids among environments.
